# Intercellular adhesion molecule-1 expression in activated eosinophils is associated with mucosal remodeling in nasal polyps

**DOI:** 10.3892/mmr.2015.3174

**Published:** 2015-01-09

**Authors:** JINGWEI XIN, HUI SUN, HONG KONG, LIN LI, JUN ZHENG, CHUNXIA YIN, YANG CAO, YUNXIAO JIA, CHAOXU LI

**Affiliations:** 1 Department of Otorhinolaryngology-Head and Neck Surgery, China-Japan Union Hospital, Jilin University, Changchun, Jilin 130033, P.R. China; 2 Department of Thyroid Surgery, Jilin Provincial Key Laboratory of Surgical Translational Medicine, China-Japan Union Hospital, Jilin University, Changchun, Jilin 130033, P.R. China; 3 Department of Gynecology and Obstertrics, Changchun Obstetrics-Gynecology Hospital, Changchun, Jilin 130042, P.R. China; 4 Department of Cerebral Surgery, The People’s Hospital of Changchun, Changchun, Jilin 130051, P.R. China

**Keywords:** airway remodeling, eosinophil, eosinophil cationic protein, epithelial damage, intercellular adhesion molecule-1, nasal polyps, remodeling

## Abstract

Nasal polyposis (NP) is characterized by chronic mucosal inflammation with infiltrating eosinophils. Eosinophil-mediated tissue remodeling may be involved in NP pathogenesis; therefore, improved understanding of tissue remodeling may result the identification of novel pathways and therapeutic strategies. The present study aimed to investigate the pathological changes occurring during tissue remodeling in NP, in order to assess the role of intercellular adhesion molecule-1 (ICAM-1) in localized tissue remodeling and the potential association between ICAM-1 expression and markers of eosinophil activation. A total of 28 eligible patients and 10 healthy controls participated in the current study. Nasal mucosal tissues of these subjects were retrospectively evaluated for mucosal remodeling using histopathological staining. ICAM-1 and eosinophil cationic protein (ECP) expression levels were determined by immunohistochemical analysis. Compared with the healthy controls, all the specimens from NP patients presented substantial epithelial damage, skewed cellular distribution with a reduced density of goblet cells, an increased density of subepithelial gland and increased subepithelial collagen deposition. In addition, the NP specimens exhibited significantly higher eosinophil infiltration and ICAM-1 expression compared with the controls. Positive correlations were observed between ICAM-1 and ECP expression levels (P=0.010), as well as between extracellular collagen deposition and ICAM-1 (P=0.010) and ECP (P=0.012) expression levels in the NP specimens, but not in the control specimens. Morphological evidence demonstrated eosinophil-mediated tissue remodeling in NP tissues. ICAM-1 expression in activated eosinophils was associated with NP remodeling, indicating the possibility that ICAM-1 may regulate NP remodeling.

## Introduction

Nasal polyposis (NP) is a common otorhinolaryngological disease, with prevalence rates in the worldwide population in the range of 2–4%; however, the disease is difficult to treat (1). Although specific etiological factors for NP have been proposed, including allergies, infections and inflammatory reactions, the pathogenesis of the disease remains to be elucidated (1). Previous studies have been established that predominantly eosinophilic inflammation is involved in NP pathogenesis (2–6). In addition, several cell-selecting and activating chemokines and adhesion molecules have been demonstrated to be important in NP development (2,7,8), although their impact and contribution to disease persistence and recurrence remains unclear.

Tissue remodeling often occurs in response to inflammatory conditions and can result in localized normal reconstruction or pathology. Although a certain degree of remodeling is expected to occur with all inflammatory diseases, its regulation is mainly disease-specific (9–12). Abnormal airway remodeling is considered to be associated with asthma and has been demonstrated to present epithelial damage, increased blood vessel cross-sectional area, airway smooth-muscle hyperplasia and hypertrophy, mucous gland and goblet cell hyperplasia and increased collagen deposition (13–16). Although NP, asthma and allergic rhinitis frequently occur concurrently (17), whether nasal and bronchial epithelia are afflicted by a common pathology is unclear.

The authors of the present study have previously demonstrated that eosinophil infiltration is associated with the expression and eosinophil binding of intercellular adhesion molecule-1 (ICAM-1) (2). ICAM-1 expression appears to initiate mucosal remodeling of NP; however, the upstream regulation of ICAM-1 remains to be elucidated. Several cytokine (interleukin-5, IL-5) and chemokine (eosinophil cationic protein, ECP; and eotaxin) candidates for eosinophil activation have emerged (18).

The aims of the present study were to: i) Investigate the morphological changes that occur during epithelial remodeling in NP; ii) further assess the role of ICAM-1 in localized tissue remodeling; and iii) assess the potential association between ICAM-1 expression and markers of eosinophil activation.

## Materials and methods

### Patients and tissue samples

Nasal mucosal tissue was obtained from 28 patients (male, 18; female, 10) diagnosed with NP (NP group) and admitted for functional endoscopic sinus surgery in the Department of Otorinholaryngology-Head and Neck Surgery of China-Japan Union Hospital at Jilin University (Changchun, China) between January 2008 and January 2009. The patients from which the NP specimens were obtained had a mean age of 34 years (range, 15–64 years). Tissue specimens were also obtained from 10 healthy volunteers (male, 6; female, 4) undergoing septal surgery for partial middle turbinate removal for a deviated septum (control group). The patients from which the control specimens were obtained had a mean age of 42 years (range, 14–57 years). All the healthy volunteers were non-smokers and did not suffer from infectious nasal and sinus diseases.

All the patients involved in the present study had not undergone any prior nasal surgery and were not administered any antihistamines, antibiotics, topical steroids or oral steroids within the month preceding the surgery. The NP population was limited to cases with eosinophilic inflammation. A mean measurement from five observed fields in the subepithelial area identifying ≥350 eosinophils per microscopic field (magnification, ×400) was considered to be indicative of eosinophilic inflammation. Patients with a history of asthma and allergic rhinitis, which was confirmed by a skin prick assessment and measurement of total and specific immunoglobulin E expression, were excluded from this study. The diagnosis of NP was based on the endoscopic findings, nasal sinus CT scan, clinical history and symptoms and was confirmed by post-surgical pathological examination. The interval between NP diagnosis and surgical treatment was <1 month in all cases. All the patients provided written consent prior to surgery allowing the use of their biological specimens in biomedical research. The procurement experiments were approved by the Ethics Committee of China-Japan Union Hospital, Jilin University.

### Histopathological stains

The surgically procured tissue specimens obtained from the NP and control groups were formalin-fixed and paraffin-embedded. Sections (3 μm) were stained with the following: Hematoxylin and eosin (H&E; Beijing Biosynthesis Biotechnology Co., Ltd., Beijing, China), used to quantify the density of infiltrating eosinophils [number within the field of view (FOV) at magnification of ×400] and the percentage (area-wise) of damaged epithelium; alcian blue-periodic acid-Schiff (AB-PAS; Beijing Leagene Biotech Co., Ltd, Beijing, China), used to calculate the density of goblet cells in the epithelium (number per mm^2^ of basement membrane in FOV at magnification of ×400) and the density of subepithelial glands (number per mm^2^ of basement membrane); and Masson’s Trichrome (MT; Beijing Leagene Biotech Co., Ltd), used to determine the thickness of the basement membrane and subepithelium collagen. The extent of epithelial damage and basement membrane thickening was categorized using the staging system described by Ponikau *et al* (3). A total of five random FOVs were evaluated for each stain at a magnification of ×400 by two experienced pathologists.

### Immunohistochemical analysis

ECP and ICAM-1 were detected by the streptavidin-peroxidase (SP) method using an SP (mouse) kit (Wuhan Boster Biological Technology, Ltd., Wuhan, China), according to the manufacturer’s instructions. Briefly, formalin-fixed, paraffin-embedded tissue sections (3 μm) were deparaffinized, rehydrated and pretreated with 5% normal rabbit serum (Beijing Biosynthesis Biotechnology Co., Ltd.) prior to antigen retrieval in citrate buffer (pH 6.0) at 95°C for 10 min. Next, the sections were incubated with the following polyclonal rabbit anti-human primary antibodies overnight at 4°C: ECP, diluted 1:100 in phosphate-buffered saline (PBS; USCN Life Science Inc., Houston, TX, USA); and ICAM-1, diluted 1:200 in PBS (Beijing Biosynthesis Biotechnology Co., Ltd.). Signal detection was performed using an SP kit. Negative controls were treated following the aforementioned procedure, without addition of primary antibodies. In addition, positive controls were treated following the aforementioned procedure, using previously characterized tissue specimens known to express the target antigen rather than the experimental specimens. The number and percentage of ECP and ICAM-1 immunopositive cells were assessed by two independent observers (magnification, ×400), with a bright field microscope (CX31; Olympus Corp., Tokyo, Japan) using a double-blind experimental procedure. Immunoreactivity values are expressed as the calculated mean of 10 randomly selected FOVs for each specimen. The means were calculated from the incorporated values reported by the two independent reviewers.

### Statistical analysis

Data are presented as the mean ± standard deviation. The normal distribution was verified using the Kolmogorov-Smirnov test (approximation method). Differences between the NP and control group mean values were assessed using Student’s t-test. Correlations between the infiltration and activation of eosinophils, ICAM-1 and ECP expression and the indices of epithelial damage and collagen deposition were assessed using Pearson’s analysis. P<0.05 was considered to indicate a statistically significant difference. All the statistical analyses were performed using the SPSS 13.0 software (SPSS, Inc., Chicago, IL, USA).

## Results

### Morphological characteristics of mucosa remodeling in NP

**Figure 1:**
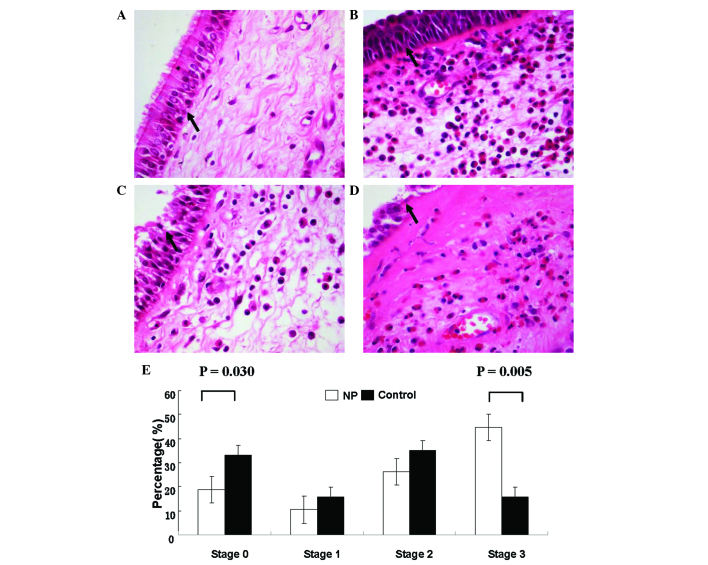
Histopathological characteristics of epithelial damage of nasal mucosa and thickening of the basement membrane was assessed in hematoxylin and eosin stained sections using the following staging system: (A) Stage 0, normal epithelium (arrow) and basement membrane; (B) stage 1, cilia loss (arrow) and basement membrane thickening; (C) stage 2, eroded epithelium (arrow), thickening basement membrane and intact basal layer; and (D) stage 3, complete erosion (arrow) of the epithelium and massive thickening of the basement membrane (magnification, ×400). (E) Percentage of NP and control specimens observed at each stage. The percentage of NP specimens exhibiting stage 3 epithelial damage indicative was significantly greater compared with the control group (P=0.005). The control group exhibited more intact epithelia or mild epithelial shedding (stage 0) compared with the NP group (P=0.030). NP, nasal polyposis.

Using the staging criteria of Ponikau *et al* (3), damage was detected in the epithelial layers of the NP and control groups (refer to Fig. 1 for a description of the four stages and representative micrographs). However, as determined from the H&E-stained micrographs, the mean percentage of the nasal mucosa that exhibited stage 3 epithelial damage was higher in the NP specimens (44.58±7.50%) compared with the control specimens (15.83±3.67%; P=0.005). By contrast, the control group specimens exhibited greater stage 0 intact epithelia and mild epithelial shedding (33.09±7.53%) compared with the NP group controls (18.75±4.44%; P=0.030; Fig. 1). However, the extent of stage 1 and 2 epithelial damage in NP specimens (10.42±3.21% and 26.25±4.66%, respectively) was found to be similar to that in control specimens (15.83±5.34% and 35.25±5.70%, respectively; P>0.05).

**Figure 2:**
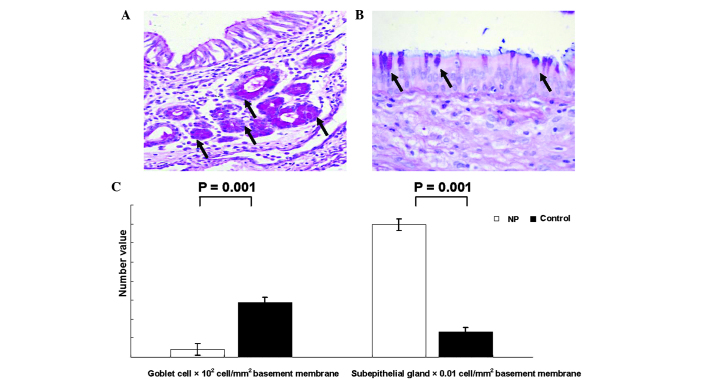
Distribution of goblet cells and subepithelial glands in NP and control nasal mucosa specimens. Alcian blue-periodic acid-Schiff staining revealed the presence of (A) clusters of subepithelial hyperplasia glands (arrows) that were distributed asymmetrically in the NP specimens (magnification, ×400) and (B) normal mucosa with pseudostratified columnar ciliated epithelium and scattered goblet cells (arrows) in the control specimens (magnification, ×400).(C) Densities of goblet cells and subepithelial glands in NP and control specimens. NP, nasal polyposis.

As shown in Fig. 2, AB-PAS staining revealed that the NP specimens presented a markedly reduced density of goblet cells (800.00±298.1 cells/mm^2^ basement membrane in FOV) when compared with control specimens (5,750.00±462.91 cells/mm^2^ basement membrane in FOV). In addition, NP specimens exhibited a greater density of epithelial glands (1.39±0.33 glands/mm^2^ basement membrane in FOV) compared with the control group specimens (0.26±0.09 glands/mm^2^ basement membrane in FOV; P=0.001). The nasal mucosa of NP specimens exhibited an asymmetric clustering of subepithelial hyperplasia glands, which was not observed in the control specimens (Fig. 2).

**Figure 3:**
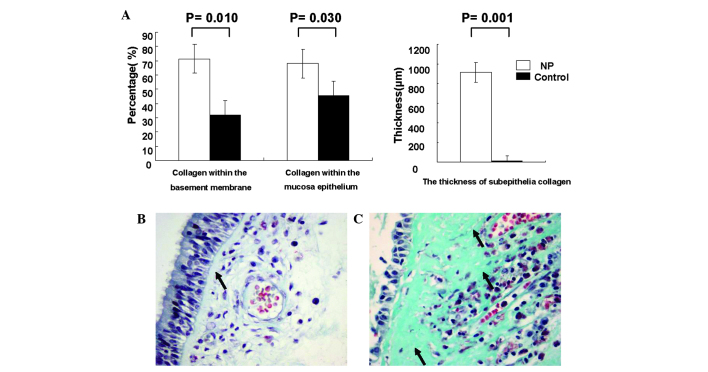
Histopathological characteristics of collagen deposition in the mucosal epithelium of NP and control nasal mucosa specimens, observed using Masson’s trichrome staining. (A) The basement membrane thickness (P=0.010), collagen deposition in the mucosal epithelium (P=0.030) and collagen thickness within the subepithelial layer (P=0.001) differed significantly between the NP and control groups. (B) Normal nasal mucosa of the control group exhibiting an intact airway epithelium and subepithelium with limited collagen deposition (arrow). (C) Nasal mucosa of the NP group exhibiting marked basement membrane thickening (arrows) and epithelial shedding. NP, nasal polyposis.

As indicated by MT-positive immunoreactivity, the extent of collagen deposition within the mucosal epithelium differed between the NP and control groups. The NP group specimens had a higher percentage of collagen within the basement membrane (71.25±20.31%) and throughout the mucosal epithelium (68.13±16.89%), compared with the control specimens (31.88±7.99%, P=0.010 and 45.63±7.76%, P=0.030, respectively; Fig. 3A). Furthermore, a morphological investigation revealed a thickened subepithelial collagen layer within the basement membrane in NP specimens compared with the controls, which resembled a broad band (Fig. 3B and C). The average thickness of the collagen band was significantly greater in the NP group (91.6 μm) compared with the controls (13.65 μm; P=0.001; Fig. 3A). Furthermore, the lamina propria of the nasal mucosa in NP specimens was often destroyed by collagen and, in certain cases, the thickened collagen extended beyond the lamina propria. Collagen was detected in the subepithelium of the specimens presenting the most severe epithelial damage (Fig. 3C).

### Activated eosinophil distribution and ICAM-1 expression

**Figure 4:**
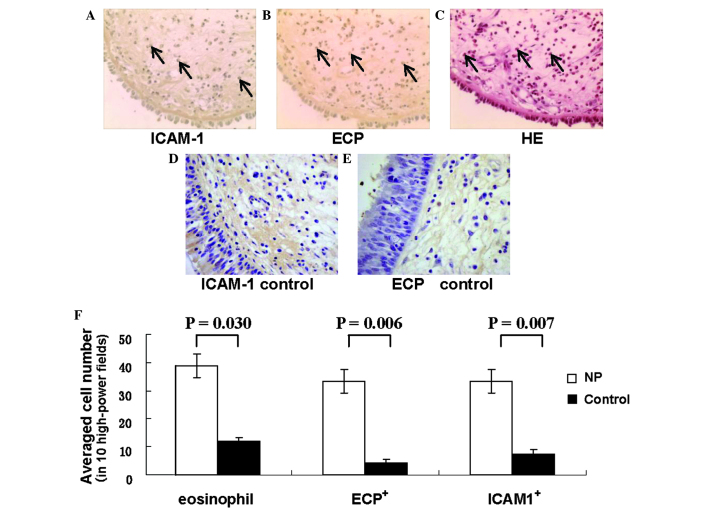
Distribution of total, infiltrating and activated eosinophils in the NP and control nasal mucosa specimens. Representative micrographs of (A) ICAM-1 and (B) ECP immunohistochemistry (brown stain) in NP specimens (magnification, ×200), revealing a large number of ICAM-1^+^ and ECP^+^ cells (arrows). (C) A representative micrograph of hematoxylin and eosin-stained NP specimens depicting marked eosinophil infiltration (arrows) of the nasal mucosa (magnification, ×200). Representative micrographs of negative control for (D) ICAM-1 and (E) ECP immunohistochemistry (magnification, ×400). (F) Distribution of total, ECP^+^ and ICAM-1^+^ eosinophils in NP and control groups. NP specimens exhibited higher cell counts for total eosinophils (P=0.030), ECP^+^ cells (P=0.006) and ICAM-1^+^ cells (P=0.007) than did control specimens. NP, nasal polyposis; ECP, eosinophil cationic protein; ICAM-1, intercellular adhesion molecule-1.

In NP specimens, the number of total (P=0.030), activated (immunopositive for ECP, ECP^+^; P=0.006) and ICAM-1 immunopositive (ICAM-1^+^; P=0.007) eosinophils was significantly greater compared with the control turbinate tissue specimens (Fig. 4). In addition, the mean number of eosinophils in the NP group (39.00±4.24 per FOV) was 3-fold higher compared with the control group (11.90±5.24 per FOV), while the mean number of ECP^+^ eosinophils in the NP group (33.58±8.32 per FOV) was almost 8-fold higher compared with the control group (4.13±1.36 per FOV; Fig. 4D). A similar trend was observed for the number of infiltrating eosinophils, with the mean number of ICAM-1-expressing eosinophils in the NP specimens (33.57±10.937 per FOV) being 4-fold higher compared with the control specimens (7.625±0.92 per FOV). In the NP specimens, infiltrating eosinophils were found to be located primarily around the vessels and glands within the subepithelial layer (Fig. 4C). Eosinophil activation (ECP^+^ eosinophils/total H&E-stained eosinophils) in the NP specimens (86.10%; 33.58/39.00) was elevated when compared with the control sections (34.71%; 4.13/11.90; Fig. 4B. Analysis of the serial sections confirmed that the majority of ICAM-1^+^ cells exhibited eosinophil morphological characteristics (Fig. 4A–C).

The extent of ECP^+^ cells in the nasal mucosal epithelium of the NP group correlated moderately with epithelial damage (r=0.424, P=0.038) and weakly with basement membrane thickness (r=0.325, P=0.012). The density of ICAM-1^+^ cells in the nasal mucosa of NP specimens correlated markedly with ECP^+^ cell density (r=0.739, P=0.010) and basement membrane thickness (r=0.603, P=0.010).

## Discussion

Nasal polyposis is a chronic inflammatory disease of the sino-nasal mucosa, characterized by edema, the infiltration of inflammatory cells, hyperplasia of the fibrous tissue, and vascularization(15,19,20). In the present study, tissue sections of NP and normal nasal mucosa were investigated for evidence of morphological changes and remodeling. Epithelial damage, basement membrane thickening and deposition of subepithelial collagen were increased in the NP specimens when compared with the healthy control specimens. Furthermore, positive correlations were observed between tissue remodeling indicators and the expression levels of ICAM-1 and ECP in NP nasal mucosa.

Mucus secreted by epithelial goblet cells is an important component in the maintenance of nasal physiological function and serves as a form of non-specific defense (21,22). However, epithelial cells, which are located on the outer layer of the nasal mucosa, are also capable of exhibiting an immune response, including the release of arachic acid derivatives, polypeptidases, matrix proteins, cytokines and other immune-associated substances (4,14). In the present study, severe damage was observed on the epithelium of the NP specimens, which included the absence of cilia and erosion of upper epithelial cell layers. These findings may be a result of increased accumulation of eosinophils and other inflammatory cells during nasal polyposis pathogenesis. Such inflammatory cells are capable of generating cytotoxic proteins, oxygen free radicals, proteinases and metalloproteinases, which may affect ciliary motor function of epithelium mucosae, as well as induce direct damage to the epithelium (13,23) through sodium and chloride channel dysfunction and eventual cellular edema (24). Epithelial damage is associated with increased epithelial shedding as a result of decreased adhesive power between the epithelium and basement membrane (15,23).

Tos *et al* (21) observed that the median density of goblet cells was 3,450 cells/mm^2^ in the anterior region of the nose, compared with 6,050 cells/mm^2^ in posteriorly located polyps. In addition, Kitapçi *et al* (22) identified that the goblet cell numbers were significantly higher in NP specimens compared with control specimens. By contrast, Malekzadeh *et al* (25) reported decreased subepithelial gland areas in NP specimens. In the present study, lower numbers of goblet cells and higher numbers of subepithelial glands were detected in the NP specimens compared with the healthy controls. Goblet cell number reduction has been previously associated with metaplasia (26,27); therefore, the presence of additional glandular tissue in the current study is consistent with the possibility of metaplasia occurring in the specimens. However, the presence and extent of cell type conversion, as well as the mechanism of goblet cell reduction, in NP remain to be elucidated. The increase in subepithelial glands may be a compensatory mechanism in response to reduced goblet cell counts and abnormal mucus secretion (13,15,24). The coupled decrease in goblet cell density and increase in supepithelial gland density reported in the present study may have resulted in the formation of a defective mucus blanket, which may in turn have led to ciliary structural damage and disrupted the directional movement of the mucus blanket, resulting in cumulative effects. In addition, exogenous physical, chemical and biological stimulants may also have contributed to epithelial mucosa damage if they were averted from the nasal tunical mucosa. This hypothesis is plausible, considering the tissue’s nested anatomical position within the ingress of the respiratory tract.

The extracellular matrix provides a crucial scaffold for epithelial organization and its deposition serves as a keystone during tissue remodeling. Deposition of the extracellular matrix by fibroblast or myofibroblastic cells in NP tissue increases epithelial mechanical strength, thereby averting tissue edema and curtailing expansion of blood vessels and glands (3,15,20). Extracellular matrix regulation in NP tissue has received scholarly attention (28,29), particularly in relation to matrix metalloproteinase 9 (MMP-9) activity. MMP-9 degrades the extracellular matrix and is involved in tissue remodeling; MMP-9 gene polymorphisms have been demonstrated to affect susceptibility to the development of chronic rhinosinusitis with NP in Chinese patients (30). Several studies have supported the hypothesis that MMP-9 is important in eosinophil-selective migration into NP tissue (2,24,29); however, Kahveci *et al* (28) did not identify a correlation between MMP-9 and eosinophils. Therefore, NP severity and recurrence may involve a mechanism other than MMP-9 (29,31).

ICAM-1, which is expressed widely in the ciliated epithelium, basal cells, submucosa, vascular endothelium and inflammatory cells, is important in the eosinophil-selective migration out of blood vessels, local aggregation and eosinophil infiltration (7). The expression of ICAM-1 correlates with nuclear factor-κB (NF-κB) signaling (32). Furthermore, upregulation of MMP-9 expression through activation of the NF-κB signaling pathway has been described in numerous diseases, including bronchial epithelial cells in asthma (32,33)

To the best of our knowledge, no systematic and anatomical investigations of collagen deposition in NP tissues exist. The data presented in the current study indicated that collagen, the principal component of the extracellular matrix, accounts for 71.25% of the basement membrane and 68.13% of the subepithelial area in NP tissues. Compared with healthy control tissues (31.88% of basement membrane and 45.63% of subepithelial area), the collagen content in NP tissues presented a ~2.2-fold and 1.5-fold increase, respectively.

Morphological examination under a light microscope revealed band-like thickening of subepithelial collagen in NP tissues, but not in control specimens. Therefore, the present study hypothesized that the observed excessive collagen generation, basement membrane thickening and subepithelial collagen banding pattern are indicative of extracellular matrix deposition in NP. In addition, the normal presentation of fibrin layers in reticular arrangements, beneath the basement membrane and within the lamina propria adjacent to the periosteum, was not observed in the NP specimens, which in contrast exhibited a submucosal reticular skeleton structure that was damaged or replaced by diffusely distributed collagen fibers. In certain cases, thickening collagen filled the lamina propria adjacent to the periosteum. A positive correlation between the extent of the observed nasal epithelial cell damage and the subcutaneous collagen thickness was also identified in the present study. Therefore, direct exposure to bacteria, viruses, external toxins and other types of super-antigen following epithelial damage (6), was hypothesized to result in stimulation of the basement membrane and subsequent collagen hyperplasia in the present study. In addition, submucosal mesh stent structural damage may be a characteristic of tissue remodeling in NP.

Local eosinophil infiltration and activation are classical hallmarks of nasal polyp formation and development (5,24,34). Although cases of NP without eosinophilic inflammation have been described (35,36), the present study included only cases with eosinophilic inflammation. Eosinophil activation has been previously demonstrated to be associated with epithelial damage and subepithelial collagen thickness (5,6); however, the results of the present study revealed only moderate correlations between markers of tissue remodeling and indicators of eosinophil infiltration (such as ICAM-1) and activation (such as ECP). Elucidation of tissue remodeling associations may be confounded by the employment of multiple regulators and pathways. Activated eosinophils release a variety of proteins and cytokines, including ECP (37), transforming growth factor-β1, fibroblast growth factor-2, MMP-9, tissue inhibitor of metalloproteinases 1, interleukin (IL)-13 and IL-17, which may contribute to the ultimate epithelial damage and collagen deposition that occurs (19,29,30). In addition, ICAM-1 has been revealed to be expressed in vascular endothelial cells and be active in the migration of eosinophils from blood vessels to tissues (2,7). In the present study, morphological investigation under a light microscope revealed the expression of ICAM-1 in activated eosinophil cells, indicating that, in addition to its role in eosinophil-selective transmembrane migration, ICAM-1 may also be directly involved in eosinophil activation through mechanisms that remain unclear. Therefore, eosinophil activation and ICAM-1 expression may contribute to, rather than govern, the initiation of tissue remodeling in NP.

In conclusion, the present study observations indicated that eosinophil-mediated tissue remodeling may play a major role in NP pathogenesis. Although NP remodeling is often irreversible, identification of key factors, including ICAM-1 expressing eosinophils, may lead to new preventive and therapeutic strategies in combating NP recurrence. For instance, in the case that ECP or ICAM-1 are involved in NP pathogenesis, patients with NP may benefit from anti-ECP or anti-ICAM-1 therapies. Further investigation is required to identify additional regulators and elucidate the mechanisms of action in NP tissue remodeling, including the potential role of the NF-κB-inducing molecule, ICAM-1 (8,34).
